# Hahnemann’s Cautions

**Published:** 1898-05

**Authors:** Guy E. Manning

**Affiliations:** San Francisco, Cal.


### HAHNEMANN'S CAUTIONS.

GUY E. MANNING, M. D., SAN FRANCISCO, CAL.

The Words of a Novice as Representing the Thoughts of a Master.

And now abideth sycosis, syphilis, and psora, these three; but the greatest of these is psora.

These words, slightly altered as they are from the original, might close Hahnemann's powerful argument upon chronic diseases, just as the original words ended a forcible sermon of St. Paul upon charity.

We have had demonstrated that all chronic conditions especially are due to one of three causes—sycosis, syphilis or psora, and that the last is responsible for the greatest and most extended diseases. The only cure for these conditions are homœopathic remedies, rightly chosen, rightly given, and for the "greatest of these" we must rely particularly upon antipsoric remedies—*i. e.*, "those remedies which in their provings produce symptoms corresponding to those developing from psora."

We have been taught how to select a remedy homœopathically, and have seen that we must not be influenced by the name of the disease in preference to its manifestations. We have already received our warnings concerning these matters, and yet, Hahnemann knowing so well how easy it would be to be influenced by other conditions, cannot leave the subject without further caution.

Hahnemann must truly have been inspired; he must have had the power of foreseeing events; a sixth sense must have been his. Therefore, he could see where flaws might be picked in his doctrines; knowing where his followers might stumble or hesitate; never doubting for a moment the law of *Similia similibus curantur*, never faltering in his doctrine of chronic diseases, never looking backward after putting his hand to the plow, yet he could foresee for us those points in which his followers might be assailed, where we might falter before the enemy. Who of us but needs these cautions? Who of us, no matter how good a homœopath, but requires them before him? And we have them here from the master hand—the fountain head. Let them act as sign-boards, as guide-posts, placed every few miles upon our journey's way, to keep us in the straight and narrow road, and let them be written, not in fine, uncertain type, but in letters bold and large, so that he who runs may read, and let us not become so familiar with these sign-boards that we may hurry by them without reading each and every one of them.

Let them be our commandments engraved upon our hearts to guide us in our daily prescribing.

Commandment I.—Thou must not become anxious over each new or unreliable symptom, but thou must permit a properly chosen remedy to exhaust its action without addition or change.

Commandment II.—Thou shalt select the remedy carefully, considering mind, body, and soul, choosing that remedy which covers in similarity the symptoms.

Commandment III.—Thou shalt not too hastily renew the remedy.

These are the three commandments as I would derive them from the cautions, though in their natural order I would make the second number one, and follow it with the first and third.

Our patient has been closely questioned, symptoms obtained, arranged, the case studied, the similia decided upon, the antipsoric given, but now what? Is that all? No! we need further advice as to our future treatment. Our patient is not so well. So far we have acted correctly and wisely, but to-morrow, or next day, or even later, a diarrhœa develops, or on awakening he finds a soreness of the throat, or perhaps a headache annoys him. Are we then to leave our antipsoric remedy which we arrived at after so much study and which has been given properly? No, indeed; stick to your remedy, and this forms your first caution, viz.: Do not ordinarily interfere with the action of the remedy by introducing a new drug action for fleeting symptoms. Why not meet these conditions if such exist? Why not ease these, or remedy those, and then return to our original systematic antipsoric?

Because these symptoms are probably an excitation produced by the remedy itself, and on further questioning you will discover that these same things have been going on for some time at periods more or less remote, and their return proves the effectiveness of the remedy in its getting to the seat of the trouble. Let it alone then to continue its action free and untrammeled; to correct not only the present symptoms, but also those of the past.

But if on investigation these symptoms have never occurred, they are present for the first time, then they are peculiar to the medicine and not to the disease. Now these will probably prove trifling, will disappear very shortly, and the good results of the drug continue. But if they do not disappear, becoming more intense in character, then comes the exception to the caution, and our conclusion must be that we have not selected the proper homœopathic remedy. Its action must then be stopped, which we can do by antidote to destroy these symptoms, or if no known antidote, then by some other antipsoric remedy to meet these conditions more accurately.

Again, you may find what may be called a homœopathic aggravation," *i. e.*, an increase in the severity of the most prominent symptoms. This, if becoming less in severity later on, denotes a cure and a proper selected remedy; but if it continues with same strength, or even increased severity, then you may be sure that your dose of medicine was too large, and you are having a drug disease which will be similar to the disease itself, or as expressed by Hahnemann himself, "The medicine in its present intensity unfolds also all its other symptoms which annul the similarity; it produces a dissimilar chronic disease instead of the former, and, indeed, a more severe and troublesome one, without extinguishing the old original one." Like the man in the Scriptures, the last state of this man is worse than the first, for now he has two devils instead of one—the chronic psora itself, plus the drug disease.

Whether the dose is too large, or whether the drug effects are going to be produced, should be evident within three weeks, and if so, then antidotal measures or other antipsoric medicines must be supplied, remembering at the same time something of the susceptibility of the patient to the last medicine. But because the drug produced its effects upon the person on account of the largeness of the dose do not argue that you must make a change in the medicine, nor that it cannot be given again. If you are confident it is the proper similia, then most certainly give it again, but in a higher attenuation, or in speaking of it as far as the amount of medicine is concerned, in a smaller dose.

Hahnemann in this place says nothing concerning any particular dilution. He is more particular to impress upon us the caution against changing the remedy, allowing it otherwise to act without change or interference, but he does not state "that nothing is lost if the dose is given even smaller than I have prescribed. It can hardly be given too small, if only everything in the diet and the remaining mode of life of the patient which would obstruct or counteract the action of the medicine is avoided." He also favors a small dose, from the fact that if at any time a wrong choice in the remedy has been made it can be more easily counteracted.

Thus ends the first caution, a warning against changing the remedy for each developing symptom, which would thus give no drug an opportunity to act and produce a constant dosing, antidoting and re-dosing. This is undoubtedly a cause of many of our failures, and the caution is well deserved. This we may term a caution against changeability. Remember this caution number one, ye followers of Hahnemann, when in your prescribing you are inclined to see-saw, as it were, or like a butterfly, flit from one flower to another without the full extract of any! If you have found the right antipsoric remedy you *may* have the development of some previous psoric symptoms, you *may* have an increase in the severity of the symptoms, or you *may* have the drug effect, but that does not prove you have selected the wrong remedy.

The first two conditions, if they do not increase in frequency or severity, rather prove the correct choice of your remedy. If they should increase it means a waste of time and a necessity for antidotal measures, or the selection of a new remedy. The third condition argues too much of a good thing. Your remedy is correct, rightly chosen, but wrongly given, and your dose must be decreased. In fact, it is a question with me if the whole caution is not intended especially as a warning against too large doses, for Hahnemann in closing the article says: "The physician can, indeed, make no worse mistake than first to consider as too small the doses which I (forced by experience) have reduced after manifold trials, and which are indicated by every antipsoric remedy; secondly, the wrong choice of a remedy, and thirdly, the hastiness which does not allow each dose to act its full time."

Now to the second caution, which might be termed a caution against laziness and carelessness. These two sins, for sins they are, and sins they easily become if not early curbed or throttled, should be furtherest from a physician's makeup, and should be especially wanting in a homœopath. Our prescribing is such that we can ill afford to permit such a plant to obtain a foothold.

With us each case is a law to itself. There is no royal road to our prescribing, no lightning express to travel it; it is slow plodding, often a laborious journey. Each time we travel over it the trees must be felled, the rocks scattered, the way cleared. In obtaining the symptoms, in arriving at a true understanding, in delving into the past history, in searching out the family record, in classifying the points according to their prominence, in fitting the remedy to the symptoms, one case is no more like another than I am like my fellow-man. My hair may be of the same auburn shade, my mouth be fully as large, my nose follow the same Grecian curve, but my disposition, qualities, virtues, manners be as different as night and day. So with our patients in searching for their similia. Both cases may have pneumonia of same lung, in exactly the same spot, with equal severity, but time, restlessness, aggravations, ameliorations and disposition will make, as you well know, all the difference imaginable in your choice of remedy. In fact, we might have things so evenly balanced in regard to their symptoms till a mental condition, for instance, proves the hair whose weight sends the scale up with a bound. Thus I wish to impress upon you the carefulness necessary in obtaining symptoms.

How is it with the other side? With them pneumonia is pneumonia, be it right or left; pleurisy is pleurisy, whether the pain is better or worse on breathing; bronchitis is still bronchitis, asthma remains asthma, whether worse at night or the contrary condition. And the treatment will be practically the same, Morphine for pain, antipyretics for fever, expectorants for inability to raise discharge, diuretics to increase action of kidneys, laxatives or cathartics to drive the bowels to further action, or an astringent to act as a plug in case of diarrhœa, a stomach tube for indigestion, and so on.

Here comes the easy part in prescribing. Their great difficulty lies in diagnosis, and they will spend more time in determining whether the kidney is wandering aimlessly around the abdominal sea or anchored securely in the harbor designed by Providence; whether the liver is growing north by northwest or due north, or whether the blood contains five thousand red corpuscles or has lost a couple "in the shuffle."

Now I am not decrying against diagnosis, or making light of it. Far from it! For I do not think we can know too much about our patients or the condition of their "innards," and it is a grand thing to be able to give a correct name for each disease, and to separate accurately one from another. But there is a probability of being blinded by such wisdom, or of allowing the glamour of such superior knowledge to overpower the uncertain therapeusis as practiced by them. As far as prescribing is concerned, we are, or should be more interested in the character of the pulse, the changes in the expectoration, the kind of pain, the location thereof, the effect of food, the conditions before and after urination, the color of the water, the character of the evacuations, the menstrual symptoms, the mental conditions, and many other states that might at the time seem entirely foreign to the original condition. And who will deny that after going so thoroughly into every feeling and condition, good or bad, of the patient, supplemented by our physical examination, who will deny that we are not better adapted to make a correct diagnosis than the other?

But I am wandering from the subject. My purpose is to show you how necessary for a homœopath to be exact, tireless, earnest, careful, diligent in his search for the proper remedy. To be such it is necessary to keep one's self spurred up to the point of exactness, earnestness, and love of work, or else you will find yourself backsliding, guessing at the remedy, becoming satisfied with two or three symptoms.

You probably all remember the famous "Lycopodium case" of Dr. Chapman's, and the answer or excuse that it brought forth from so many of the old school, by which they thought to belittle us, viz., that we had but a single remedy for a disease, and that they would not wish to be tied down to any such single remedy system, or words to that effect. I have often wished that they might be obliged to take a Bœnninghausen and a checking list, and go over the long list of 250 remedies, time after time, finding, perhaps, two-thirds of them indicated under each symptom. Methinks then they would return most fervent thanks that they were permitted to combine as many of their remedies as they wished into one grand and glorious whole and obnoxious dose, instead of looking for a simple remedy.

But it is this fine discrimination, this close comparing that necessitates the greatest of patience and the closest of work on our part, and that makes it the grand system it is.

But it is the desire to get rid of this labor that has produced two prominent ills of our day. Hahnemann mentions one "sin," the use of the repertory. These contain "vague hints," he says, and you will probably agree that they are but vague hints. What more can you expect? If the volume is small and compact the contents must be very vague, and if exact and complete, then they are exceedingly cumbersome, and in the end unsatisfactory, on account of their completeness, requiring as much study as a proper search for the remedy might. Take for an example of the first Lippe's *Repertory*, and of the latter Gentry's *Concordance*. Who will deny but that they are both good? The former, as we know by a pure homœopath, an almost perfect materia medicist and prescriber, careful, reliable, and yet it is only a small volume, containing only the most important symptoms. Consider the other. Four or five large volumes, divided and subdivided, reiterated, minute to the smallest degree, and yet cumbersome and unsatisfactory. Between these two are repertories of the head, of the mouth, of the chest, of the mind, of the abdomen, and each quarter section of it, of the legs and back, concerning expectoration, diarrhœa, etc., consisting of one hundred pages, one volume or ten volumes, till you consider that of making repertories "there is no end." And of what value are they? They have a value if used rightly. If we are employing the "vague hints" contained therein for a good purpose, well and good. If we are using them for a guide to direct our study and our thoughts, to start our researches in the right direction, to keep us on the right track, then they are most valuable assistants. But if we are going to prescribe from them alone, are intending to rely upon them entirely, are going to make of them an aid to laziness, then we are making a wrong use of them, and they prove a sin. They are very strong inducements to laziness, to carelessness. One is inclined to lean too heavily upon the arm of their guide, and in the same way one is apt to content himself with the repertory headings, "vague" hints that they are. These kinds of prescribers are called by Hahnemann "bunglers," because they are continually obliged to change their prescription, depending upon one or two symptoms and vague hints for their choice in remedy, instead of hunting more closely, the ailments in the meantime becoming more and more complicated by drug action, drug effect, and frequent changes. Do you know any "bunglers?" If so, do you know the cure? Let me give it to you as Hahnemann's second commandment: "Thou shalt select thy remedy carefully, considering mind, body, and soul, choosing only that remedy which covers in similarity the symptoms present." This is not conducive to laziness or love of ease, you will acknowledge, but it will be conducive to better prescribing and better results.

I mentioned that there were two causes which had much to do towards laziness and inaccurate prescribing. Do not understand that there are only two, but they are prominent ones. The second cause is the tablet. This is not a product of Hahnemann's time. It is a modern development in an attempt to improve upon our master, and I am willing to acknowledge that when we try to improve upon him we get further away from Homœopathy.

There need be nothing objectionable about the tablet if it should be made with the same carefulness and cleanliness that all our preparations are supposed to have. But such is not the case. The molds are used again and again; foreign powders are used to dust the molds, and foreign substances added to keep the shape. Does that correspond to Hahnemann's teachings or rules regarding preparation of medicines? But the strongest objection is that it favors polypharmacy, aids it, applies it.

Consider an advertised list of a homœopathic pharmacy to be ordered by numbers, and running thus:

Worms—Cina 2x, Spigelia 3x, Merc-dulc 1x.

Croup—Spongia 1x, Aconite 2x, Iodine 2x.

Diarrhœa—Aloes 3x, Merc-cor. 1x, Opium $\frac{1}{8}$ gr.

Cough—Phos., Ant-tart, Codein.

Rhinitis—Camph., Quinine, Gelsemium.

Neuralgia—Acetanilid 7 parts, Caffine 2 parts, Sod-bicarb. 1 part, and so on by the dozen.

Are they any better than any old school prescription? Is this not polypharmacy with a vengeance? Is this not doing more harm to Homœopathy than all your lectures or papers can do good? And who is at fault? Your prescriber or your pharmacy? Your so-called homœopathic physician, or your so-called homœopathic pharmacy? Strictly your former, for without the demand there would be no supply. Such things must cause the bones of Hahnemann to turn in his grave. Is it any wonder that the old school sneer at us and at our practice, call us hypocrites, or demand that we shall either drop the name or the doctrine?

The third caution is directed against haste. We have already been cautioned against changing the remedy for new symptoms until we found that they were prominent or increasing, but now it becomes necessary to warn us against renewing the remedy for the original symptoms until we are perfectly satisfied that the first dose has done its full work, and this, I believe, is the most needed of the three cautions. Who of us but are guilty of haste in the matter? This haste is perfectly excusable; it is two-sided, being present with patient as well as physician, and it is simply a desire to improve the condition which has been of such long standing, and to hurry up our cure. It is also due to our early education. We cannot find it easy to get out of our minds material things and frequent application. It is difficult for a human mind to easily grasp the thought, or to hold to it after it is grasped, that these bulky frames of ours are made up of atoms which can be acted upon by atoms, or that the effect of these atoms may extend over a continued period, for better or worse.

Our minds are not fine enough to grasp such thoughts, and we continue to pour masses down our throats frequently, because we in our ignorance and in our vanity flatter ourselves that we are large bodies of matter which only sensible matter can affect.

We forget that any sudden thought, for instance, destroys an incomprehensible small amount of brain substance; that the dead matter is taken up by a minute blood corpuscle, too small almost for our mind to imagine, and that it carries it through a whole system of vessels, passing at last into the lungs, where in vessels fine beyond comparison, with walls composed of a single layer of cells, it gives up this matter; the oxygen, in a minute molecule enters the corpuscle, and it goes on its way rejoicing.

Consider the food taken in to nourish the body, acted upon by juices and digestive ferment. It also is absorbed in very minutest particles through membranes. Our Texas steer has become a molecule of fibrin, a globule of fat, an atom of chyle taken into lymphatics and blood-vessels; atom following atom, molecule following molecule in a rapid chase for that life-giving stream, the blood, to be hurried on to those tissues crying for repair, there to be given up. How? In teaspoonful doses? By the quart or pint? No, in atoms.

This is simply to show that ordinary life is carried on by molecules. Nature acts on the atomic theory. Nature repairs by atoms, she grows by atoms, she nourishes by atoms, she acts through atoms.

But it is with the length of action that we have the most to do at this time.

Experiment has proven, if you are not already aware of the same, that medicine acts for an extended time, that its effects can be determined for several days, that different medicines act for a longer or shorter period than others, but that in none do we have a complete duration in a single day. In fact, it is well known that a single dose of medicine acts for days.

Does Opium, for instance, exhaust its sleep-producing action in a few hours? Will Strychnine fail to produce its nerve action in a day? By no means. It takes days and days for the system to throw off these severe effects. This all in a system free from any morbid influences. How much more sensitive, you are well aware, when influenced or weakened by disease. How much more susceptible to any condition; how much greater the action of the drug.

Consider for a moment how generally, how universally, the system must be affected by a miasm. From infancy, through childhood, youth, adult life, perhaps to middle age or later, this miasm has been within the system, attacking perhaps every portion of the organism, undermining all the parts, breaking out here and there with renewed vigor. Can you expect a remedy to attack such a condition at once; will it strike at the whole evil instantaneously and each organ delicate and vigorous at the same time?

In fact, Hahnemann, in his experiments, found in chronic diseases that they were long lasting, or rather the more "tedious" the psoric disease, the longer the antipsoric medicines continued their action. He also learned, and we may say discovered the contrary of this rule, that the more acute the disease the shorter the period of action, even those which in the healthy body show a long period of action.

So in this Hahnemann himself sees the necessity for, and does not decry against, the repetition of the same medicine in acute diseases, or when a disease which is chronic rises into an acute case.

This he calls the only allowable exception to the rule, but that there can be exceptions he in his wisdom foresees, and it is then that he lays down the rule that "when the peculiar symptoms of the disease to be treated, after fourteen, ten, and even fewer days, visibly cease to diminish, so that the improvement manifestly has come to a stop, without any disturbance of the mind, and without the appearance of any new and troublesome symptoms, so that the former medicine would still be perfectly homœopathically suitable, only then, I say, is it useful, and probably necessary to give a dose of the same medicine of a similarly small amount, but most safely in a different degree of dynamic potency."

He is inclined to limit those antipsoric drugs capable of immediate repetition to Hepar, Sulph., and in some cases Sep., considering it rather better practice, and usually necessary in chronic diseases, to change to another antipsoric.

We are speaking at this time particularly of chronic diseases. We give a medicine which we find upon thought or study to be antipsoric. This will probably not show its good effect for eight or ten days, and it may not create any marked response till even the thirtieth day. When the response does come the good effects continue for some time.

The healing process in a wound might form an example, a visible example, as it were. The wound without treatment gets gradually worse, suppuration begins, pus burrows. Now we add some healing dressing. No effect may be noticed for a few days, but soon a more healthy condition appears, then granulation and the response begins. Without renewing the application the healing goes on.

A cure cannot be accomplished more quickly and surely than by allowing the suitable antipsoric to continue its actions so long as the improvement continues, even if this should be several, yea, many days beyond the assigned supposed time of its duration. There may come a time, there will undoubtedly come more than once a period when these symptoms will crop up again, will reappear, perhaps in milder form; they should not appear in an aggravated manner if the medicine is the correct similia, though Hahnemann speaks of such a thing as an aggravation, a moderate homœopathic aggravation, for an hour, or even half a day, even though the medicine is acting well and rightly, even though but a single dose has been taken.

If, as has been said, this return of the original symptoms recur, and in a milder form, then you have your warning for another dose of medicine.

Now you can see why it may be termed a caution against impatience.

It will also require the closest of observation, and perhaps experiment, to note the point at which to give your second, third or succeeding dose. A difficult point in prescribing has always been to determine just the point at which a set of symptoms become drug effects and not the disease symptom. Now, in the same line, it becomes a delicate task to observe just the point at which the drug should be renewed, when the remedy has ceased to act. Hahnemann says this is decided alone by experience and careful observation, and he adds, "it always decided in my manifold exact observations so as to leave no doubt remaining."

Hear also these words of Hahnemann's:

"It is a fundamental rule in the treatment of chronic diseases to let the action of the remedy, selected in a mode homœopathically, appropriate to the case of disease, which has been carefully investigated as to its symptoms, come to an undisturbed conclusion as long as it visibly advances the cure and the while improvement still perceptibly progresses."

"This method forbids any new prescription, any interruption by another medicine, and forbids as well the immediate repetition of the same remedy.

"Where, as is usually the case in chronic diseases, antipsoric remedies are necessary, the more frequent sudden change of them is a sign that the physician has selected neither the one nor the other in an appropriately homœopathic manner, and had not properly investigated the leading symptoms of the case before presenting a new remedy. This is a frequent fault into which the homœopathic physician falls in urgent cases of chronic disease, but oftener still in acute diseases from over haste, especially when the patient is a person very dear to the heart.

"We cannot flatter ourselves that the antipsoric medicine given was rightly selected, or that it will forward the cure of a chronic disease, if it quickly and entirely destroys as if by a stroke of magic the most troublesome symptoms, old, great, continuous pains, tonic and chronic spasms, etc., so that the patient almost immediately after taking the medicine fancies himself free from suffering, as if he were already restored and as if in heaven. This descriptive effect shows that the medicine here acts antipathically, as an opposite or palliative, and that in the days following we cannot expect anything from this remedy but an aggravation of the disease."

This waiting, this impatience, has proved a stumbling-block to many. Oh! that we could have a Hahnemann to pour into our ears such words of wisdom, to stimulate our faltering hearts, to breathe into our sluggish veins, to take us from the mire of polypharmacy, of repetition, of frequent dosing, of massive quantities, of laziness and carelessness, impatience and disbelief, and raise us up to the pinnacle of true Homœopathy.

But even then, though we were raised to this point of purity and wisdom, would we not need a wall to keep us in and prevent our backsliding to those lower depths from which we have been rescued?

We have Abraham and the prophets; hear them. Did not the Jews of old put to death their Master? And though our beloved Hahnemann rose from the dead and appeared among us teaching his doctrines, would not we who bear his name likewise cry "crucify him," instead of following in his train?

